# A randomised, double-blind study of polyethylene glycol 4000 and lactulose in the treatment of constipation in children

**DOI:** 10.1186/1471-2431-14-153

**Published:** 2014-06-19

**Authors:** Suporn Treepongkaruna, Nipat Simakachorn, Paneeya Pienvichit, Wandee Varavithya, Yothi Tongpenyai, Philippe Garnier, Hélène Mathiex-Fortunet

**Affiliations:** 1 Department of Paediatrics, Faculty of Medicine, Ramathibodi Hospital, Mahidol University, Rama 6 Road, Bangkok 10400, Thailand; 2 Division of Paediatrics, Maharat Nakhon Ratchasima Hospital, Nakhon Ratchasima, Thailand; 3 IPSEN, Boulogne-Billancourt, France

**Keywords:** Constipation, Macrogol, Lactulose, Children, Stool frequency

## Abstract

**Background:**

Chronic constipation is frequent in children. The objective of this study is to compare the efficacy and safety of PEG 4000 and lactulose for the treatment of chronic constipation in young children.

**Methods:**

This randomised, double-blind study enrolled 88 young children aged 12 to 36 months, who were randomly assigned to receive lactulose (3.3 g per day) or PEG 4000 (8 g per day) for four weeks. The primary efficacy variable was stool frequency during the fourth week of treatment. Secondary outcomes were the number and frequency of subjective symptoms associated with defecation at each visit.

**Results:**

Stool frequency was comparable in the two groups at baseline (lactulose: 0.7 ± 0.5; PEG 4000: 0.5 ± 0.55). Mean stool frequency increased from 0.70 ± 0.50 stools/day at baseline to 0.80 ± 0.41 at Week 4 in the lactulose group and from 0.50 ± 0.55 to 1.10 ± 0.55 stools/day in the PEG 4000 group. A significant difference was observed in the adjusted mean change from baseline, which was 0.15 stools/day in the lactulose group and 0.51 stools/day in the PEG 4000 group, with a least-squares mean difference of 0.36 stools/day [95% CI: 0.16 to 0.56]. With respect to secondary outcome variables, stool consistency and ease of stool passage improved more in the PEG 4000 group (*p* = 0.001). The incidence of adverse events was similar in both groups, the majority of which were mild.

**Conclusions:**

PEG 4000 has superior efficacy to lactulose for the treatment of chronic constipation in young children and is well tolerated.

**Trial registration:**

US National Institute of Health Clinical Trials database; study NCT00255372 first registered 17th November 2005.

## Background

Constipation is an extremely common problem in children accounting for 3% of all visits to paediatric outpatient clinics and up to as many as 25% of all visits to paediatric gastroenterologists in the United States [1,2]. Nonetheless, the prevalence of functional constipation in the community is not known with any precision, and prevalence rates ranging from 0.7% to 29.6% have been reported in the literature, with a median of 8.9% [3]. In Asian populations, reported prevalence rates are at the higher end of the range, for example 29.6% in Hong Kong [4] and 24.9% in Shanghai [5].

The occurrence of chronic constipation in children can lead to significant abdominal pain, appetite suppression, lowered self-esteem due to faecal incontinence, social isolation, feelings of depression, school absenteeism and family disruption [6]. Moreover, if constipation in children is not adequately managed, it may persist into adulthood. On the other hand, effective early treatment in children may provide a definitive cure [7,8].

Treatment goals are to produce soft, painless stools and to prevent the reaccumulation of faeces [6], which can be achieved through dietary modification, behavioural interventions, and the use of laxatives, or a combination thereof [6]. With respect to medication, choices include lubricants, such as paraffin oil, osmotic laxatives, including lactulose, sorbitol, magnesium hydroxide and polyethylene glycol (PEG), and stimulant laxatives such as senna or bisacodyl.

Polyethylene glycol (PEG, macrogol) is a polymer of ethylene oxide units of variable molecular weight. Polymers with a molecular weight of over 3000 are essentially unabsorbed or metabolised in the intestine and are used as osmotic laxatives, due to their high water binding capacity [9]. Two PEG preparations, PEG 3350 (*Glycolax*®, *Miralax*®, Braintree Laboratories Inc, Braintree, Mass, USA, *Transipeg*® Bayer) and PEG 4000 (*Forlax*®, Ipsen, France) have been developed for this purpose.

Although the superiority of PEG over other osmotic laxatives has been well documented in adults, the evidence base is more restricted in paediatric populations. The number of well-designed randomised, double-blind clinical trials that have evaluated PEG in the management of chronic constipation in children remains relatively limited, and the number of subjects evaluated is low [10]. These include two placebo-controlled studies of PEG 3350 [11,12], three studies comparing PEG 3350 to lactulose [13-15], two comparing PEG 4000 to lactulose [16-18], one comparing PEG 3350 to magnesium hydroxide [19], two comparing PEG 4000 to magnesium hydroxide [20,21] and two comparing PEG 3350 to liquid paraffin oil [22,23]. The majority of these studies included older children and little data is available in children younger than three years of age. Further clinical trials would be helpful to extend the available evidence base, in particular studies performed in young children. The primary objective of the present study was to compare the efficacy of PEG 4000 to that of lactulose in the treatment of young children aged between 12 to 36 months with chronic constipation.

## Methods

This phase III randomised, double-blind, active-controlled, parallel-group study was conducted in outpatients consulting in two general hospitals in Thailand (Ramathibodi Hospital, Mahidol University, Bangkok and Maharat Nakhon Ratchasima Hospital, Nakhon Ratchasima) from 2004 to 2008. The study was registered in the Clinical Trials database of the US National Institute of Health under the study identifier NCT00255372.

Each patient underwent four study visits. At the screening visit (Visit 1; Week -2), inclusion criteria were verified and demographic and clinical information was documented. The patient’s family was provided with dietary advice to restore normal bowel movements and with a diary in which stool output was to be recorded. At the inclusion visit (Visit 2; Week 0), if constipation had not resolved through dietary modification, eligibility criteria were verified and the patient was randomised to one of the two treatment groups. A new stool diary was provided. The patients attended two follow-up visits (Visits 3 and 4; Weeks 2 and 4) to document efficacy and safety of treatment.

### Patients

The study included young children aged between 12 to 36 months with a diagnosis of chronic functional constipation based on a modification of the Rome II criteria for infants and preschool children [24]. This was defined as EITHER a stool frequency of ≤2 per week persisting for at least three months OR the presence of pebble-like, hard stools, painful defecation or faecal incontinence for at least three months. Faecal incontinence was defined operationally as soiling of underclothes in children who had already acquired toilet skills.

All patients were followed for two weeks (between Visits 1 and 2) following provision of dietary advice, and only those children whose symptoms failed to improve during this period were eligible. Children whose parents failed to provide written informed consent were not eligible. Exclusion criteria included the presence of organic bowel disease, suspected gastrointestinal obstruction, a history of GI surgery, any other condition or baseline finding that might, in the opinion of the investigator, interfere with the implementation or interpretation of the study, and a history of hypersensitivity to the investigational drug or related drugs.

In order to evaluate possible inclusion bias, each investigator documented in a screening log all patients who were considered eligible for the study but who were not in fact enrolled. For each patient, the primary reason for exclusion was recorded. Patients could be withdrawn from the study if their parents requested discontinuation of treatment because of lack of efficacy.

### Treatment

Treatment was allocated using a randomisation list of treatment allocation codes prepared by the contract research organisation responsible for operational management of the study. After confirmation of the eligibility criteria, patients were randomised in a sequential order within each centre. The randomisation list was kept confidential in a safe and secure location until approval was received for the study to be un-blinded for analysis.

Eligible subjects were randomly assigned to receive either lactulose (3.3 g per day) or PEG 4000 (*Forlax*®; 8 g per day) for a period of four weeks. These doses correspond to the recommended doses for use in young children provided in the prescribing information for these two laxatives. Lactulose was provided as a 3.3 g sachet dissolved in 60 mL of water taken in the morning. A sachet of lactulose placebo containing an inert powder (Glucidex IT38 and saccharin) with the same flavour as lactulose was taken in the evening. PEG 4000 was provided as a 4 g sachet dissolved in 60 mL of water taken in the morning, and an identical sachet taken in the evening. All sachets were similar in size, colour, smell, taste and appearance in order to ensure adequate blinding of the study medication. In the event that a patient did not receive the study medication as planned, the primary reason for this was documented in the case report form.

Children with faecal impaction received an enema (*Unison*®, sodium chloride 15% solution; 10 mL in one or two doses) in order to empty the rectum before starting the study treatment. Parents were permitted to give a *Unison*® enema if their child failed to have a stool for three days. Use of other laxatives or purgatives such as milk of magnesia, mineral oil or ispaghula husk was not permitted during this study.

### Data collection

At the screening visit (Visit 1) and the inclusion visit (Visit 2), the age and gender of the patient were recorded and weight, height and vital signs measured. Information was documented on medical and treatment history, and a complete physical examination was performed. At the follow-up visits (Visits 3 and 4), stool output during the preceding two-week period were identified from the patient diary. The parents were asked about the occurrence of potential adverse events.

### Outcome measures

Stool frequency was determined for Weeks -1 (baseline), 1, 2 and 3 and 4 as the mean number of stools passed per day for the seven days of the week. The primary efficacy variable was stool frequency at Week 4. Secondary efficacy measures were stool consistency, ease of stool passage and the occurrence of subjective symptoms associated with defecation, namely cramping, flatus and anal irritation at each visit. Adverse events (AEs) were assessed from discussion with the parents at Visits 3 and 4. Incidence of AEs and serious AEs (SAEs) was documented over the entire four-week study period. All AEs were coded using the NCI Thesaurus. Compliance was assessed by counting returned medication sachets. If the patient took <70% of the scheduled amount of medication intake in Week 4 or <80% over the entire treatment duration, this was regarded as a major protocol violation.

### Statistical analysis

The number of patients to be included in the study was determined through *a priori* power calculations. The anticipated on-treatment mean stool frequency was 0.9 ± 0.6 per day in the lactulose treatment group and 1.3 ± 0.7 per day in the PEG 4000 treatment group. These projections were derived from a previous comparative study of lactulose and PEG 3350 in chronic constipation in adults [25], no studies in children having been documented at the time the study protocol was designed. A sample of 42 eligible patients in each treatment group would be required to detect a difference in mean stool frequency of 40% between the PEG 4000 and lactulose treatment groups with a power of 80% at a two-sided significance level of 0.05. Assuming a drop-out rate of 16%, it was thus planned to recruit a total of 50 patients per treatment group.

Three study populations were assessed, namely a safety population, defined as all patients who received at least one dose of study medication, an intent to treat (ITT) population, defined as all patients in the safety population for whom at least one post-treatment measure of stool frequency was available, and a per protocol (PP) population, defined as all patients in the ITT population without a major protocol deviation. The primary efficacy analysis was performed in the ITT population and a sensitivity analysis in the PP population. The safety analysis was performed in the safety population.

In the case of premature study discontinuation, the last data value recorded in the patient diary was assigned according to the principle of last observation carried forward (LOCF). In the case of missing data for stool on a given day during any week, the mean of the values on other days in the same week was used to interpolate the missing one. For a given week, the mean value was computed only if at least four of the seven daily assessments of the week in question were documented.

The primary objective of the study was to detect a difference in stool frequency during the fourth week between the two treatment groups. Stool frequency during the fourth week of treatment was assessed across treatment arms using analysis of covariance (*ANCOVA*), in which site and baseline stool frequency (during Week -1) were treated as covariates. In addition, the 95% adjusted confidence interval for the treatment effect was also estimated. Interactions between treatment group on the one hand and site and baseline stool frequency on the other were estimated.

Because of potential deviations from normality of stool frequency, (as established by the Kolmogorov-Smirnov test both on raw and transformed data using log, square root and Box-Cox transformations), a *post hoc* sensitivity analysis was performed to compare the treatment effects using a generalised estimating equation model with a Poisson distribution for repeated measures, taking into account baseline stool frequency, site, treatment, study period and interactions between treatment and study period, treatment and site and treatment and baseline stool frequency.

Stool consistency and ease of stool passage were rated as change from baseline in one of three categories of change, namely 0 (harder stools/more difficult passage), 1 (no change from baseline) or 2 (softer stools/easier passage). Similarly, the occurrence of associated symptoms (cramps, flatulence and anal irritation) were rated as 0 (decrease from baseline), 1 (no change from baseline) or 2 (increase from baseline). Changes in symptom scores over time were compared between treatment groups using a generalised estimating equation model with a negative binomial distribution. Treatment effect estimates are presented as relative risks with their 95% confidence interval.

The statistical analysis was performed using SAS software Version 9.1.3 (SAS, Raleigh, USA).

### Ethics

The study was conducted according to the Declaration of Helsinki and pertinent national legal and regulatory requirements. The protocol was approved by the appropriate independent ethics committees (the Committee on Human Rights Related to Researches Involving Human Subjects, Faculty of Medicine, Ramathibodi Hospital, Mahidol University, Bangkok, Thailand and the Institutional Review Board of the Maharat Nakhon Ratchasima Hospital, Nakhon Ratchasima, Thailand). Patient confidentiality was ensured by assigning each subject a study code that was used in the case report form in place of the patient’s name. Patients were free to withdraw from the study at any time for any reason. A parent of all participating children gave their written informed consent for their child to participate in the study.

## Results

### Study population

**Figure 1:**
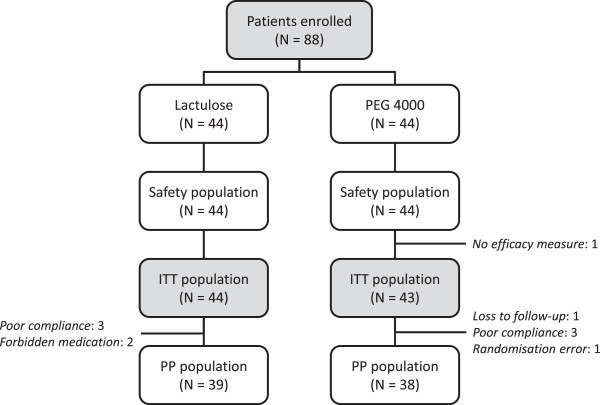
Patient flow through the study.

A total of 88 subjects were enrolled in the study, of whom 44 were randomised in each treatment arm. These constituted the safety population. One patient randomised to PEG 4000 did not complete the patient diary and was thus excluded from the ITT population, which consisted of 87 patients. Ten patients in the ITT population (five in each group) were excluded from the PP population due to major protocol violations, principally poor compliance. The flow of patients through the study is illustrated in Figure 1. The two study centres were evenly balanced (44 patients enrolled in each centre). The mean exposure to the study treatment was 29.2 ± 1.77 days [median: 29 days; range: 28 – 39 days] in the lactulose arm and 28.9 ± 5.81 days [median: 29 days; range: 4 – 56 days] in the PEG 4000 treatment arm.

The baseline socio-demographic characteristics of the study population are shown in Table 1. The mean age was 1.99 ± 0.50 years. Overall, there were more boys than girls enrolled (56.8% vs. 43.2%). All children enrolled fulfilled the stool frequency criterion for chronic constipation (≤2 stools/week) and fifteen (seven in the lactulose group and eight in the PEG 4000 group) also fulfilled the stool consistency criterion (hard stools most of the time). The mean duration of chronic constipation was 43.8 ± 25.4 weeks and 53 children (60.2%) had previously been treated for their constipation with at least one other agent, principally lactulose (22 children), sodium chloride enemas (15 children), liquid paraffin (9 children), glycerine-based suppositories (8 children) or PEG 4000 (5 children). The two study groups were well balanced. In particular, stool frequency at baseline did not differ between the two groups (*p* = 0.084; Mann–Whitney U-test).

**Table 1:** Socio-demographic and clinical characteristics of patients at the baseline study visit in the enrolled (safety) population (N = 88)

	**Lactulose**	**PEG 4000**	***p***
	**(N = 44)**	**(N = 44)**	
Gender, n (%)			
Boys	24 (54.6%)	26 (59.1%)	0.83
Girls	20 (45.5%)	18 (40.9%)	(χ^2^ test)
Age, years			
Mean ± SD	1.98 ± 0.52	1.99 ± 0.50	0.83
Median [range]	2.0 [1–3]	2.0 [1–3]	(Student’s *t*-test)
Duration of chronic constipation (weeks)			
Mean ± SD	44.4 ± 28.8	43.2 ± 21.8	0.73
Median [range]	36 [8–116]	40 [10–104]	(Wilcoxon test)
Previous treatment for chronic constipation	26 (59.1%)	27 (61.4%)	0.99 (χ^2^ test)

### Primary efficacy outcome

In the ITT population, the mean stool frequency increased from 0.7 stools/day during the baseline period to 0.8 at study end (Week 4) in the lactulose group and from 0.5 to 1.1 stools/day respectively in the PEG 4000 group (Table 2). After adjustment for stool frequency at baseline and site, stool frequency was significantly higher (*p* = 0.0005) in the PEG 4000 group than in the lactulose group during Week 4. The adjusted mean change from baseline in stool frequency was 0.15 stools/day in the lactulose group and 0.51 stools/day in the PEG 4000 group, corresponding to a least-squares mean difference of 0.36 stools/day [95% CI: 0.16 to 0.56]. No significant treatment × site (*p* = 0.13) or treatment × baseline stool frequency (*p* = 0.66) interactions were observed.

**Table 2:** Stool frequency (number of stools per day) in the ITT population (N = 87) and the PP population (N = 77)

**ITT Population**	**Lactulose**	**PEG 4000**
	**(N = 44)**	**(N = 43)**
Baseline	0.7 ± 0.5	0.5 ± 0.55
Week 4 (study end)	0.8 ± 0.41	1.1 ± 0.55
Change (Week 4 – Baseline; unadjusted)	0.1 ± 0.55	0.6 ± 0.63
Adjusted difference in mean change from baseline	0.36 [95% CI: 0.16 to 0.56]	
**PP Population**	**Lactulose**	**PEG 4000**
	**(N = 39)**	**(N = 38)**
Baseline	0.7 ± 0.51	0.5 ± 0.56
Week 4 (study end)	0.8 ± 0.39	1.1 ± 0.56
Change (Week 4 – Baseline; unadjusted)	0.1 ± 0.55	0.6 ± 0.63

**Figure 2:**
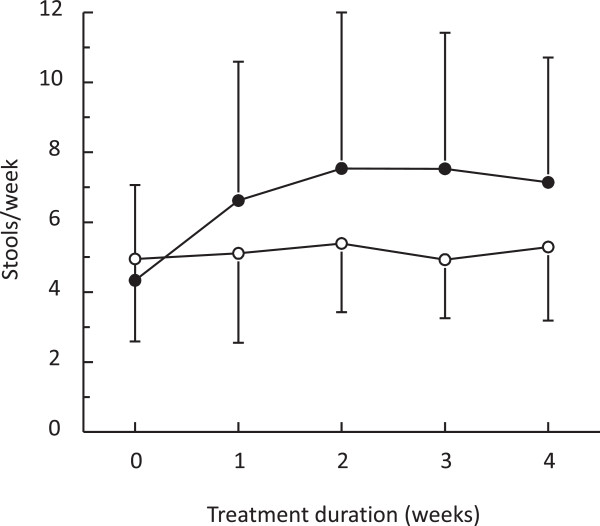
**Stool frequency by treatment week.** Data are presented as mean values ± standard deviations. Open symbols: lactulose group; filled circles: PEG 4000 group.

Since stool frequency at baseline did not follow a normal distribution using either non-transformed or transformed data (*p* <0.01; Kolmogorov-Smirnov test), a *post hoc* analysis of the data was performed using a generalised estimating equation model for repeated measures. A significant difference between the two treatment groups was also observed in this model (*p* <0.001). No treatment × site interaction was observed. The evolution of stool frequency over the treatment period is presented in Figure 2. The higher on-treatment stool frequency in the PEG 4000 group is observed at all time-points, starting from the first week of treatment.

In the PP population, a treatment × site interaction of borderline significance (*p* = 0.0511) was observed and, for this reason, the treatment effect size was estimated independently for each site. At site 1 (Bangkok), the adjusted mean change from baseline was 0.10 stools/day in the lactulose group and 0.67 stools/day in the PEG 4000 group, corresponding to a least-squares mean difference of 0.57 stools/day [95% CI: 0.23 to 0.91], which was significant (*p* = 0.0016). At site 2 (Nakhon Ratchasima), the adjusted mean change from baseline was 0.29 stools/day in the lactulose group and 0.47 stools/day in the PEG 4000 group, corresponding to a least-squares mean difference of 0.17 stools/day [95% CI: -0.087 to 0.432]. This difference did not reach the statistical significance.

### Secondary efficacy outcomes

The secondary efficacy outcomes are summarised in Table 3. With regard to stool consistency, improvements in stool consistency scores were higher in the PEG 4000 group than in the lactulose group (*p* = 0.0012), with a relative risk of achieving softer stools of 1.27. Compared to baseline, softer stools were reported at the end of the study (Week 4) for 58.6% of children. Similarly, stool passage improved to a greater extent in the PEG 4000 group than in the lactulose group (*p* = 0.001), with a relative risk of achieving easier stools of 1.35. Stool passage was reported as easier for 40.9% of children at Week 4. No between-group differences in symptom score evolution were observed with respect to cramps, flatulence or anal irritation. The proportion of children for whom the intensity of cramps was modified at the end of treatment, during Week 4 was different between the two groups (*p* = 0.02). The proportion of children with unchanged cramp intensity was higher in the PEG 4000 group than in the lactulose group, whereas the proportion of patients whose cramps had worsened, as well as those whose cramps had improved, were both higher in the lactulose group than in the PEG 4000 group. By Week 4, flatulence had improved in 24.1% of children and anal irritation in 31.0%. Four children in the lactulose treatment group required rescue treatment with a sodium chloride enema.

**Table 3:** Secondary efficacy outcome measures (ITT population: N = 87)

	**Lactulose**	**PEG 4000**	**Relative risk**	***p***
	**(N = 44)**	**(N = 43)**	**[95% CI]**	
*Stool consistency*				
Mean symptom score ± SD			1.27 [1.1 - 1.46]	0.0012
Baseline	1.16 ± 0.83	1.35 ± 0.95		
Week 2	1.71 ± 0.88	2.19 ± 0.73		
Week 4	1.71 ± 0.80	2.09 ± 0.65		
Change (W4 – baseline)				0.87
Worsened	6 (13.6%)	6 (14.0%)		
No change	11 (25.0%)	13 (30.2%)		
Improved	27 (61.4%)	24 (55.8%)		
*Ease of stool passage*				
Mean symptom score ± SD			1.35 [1.13 - 1.62]	0.001
Baseline	0.93 ± 0.95	0.98 ± 0.77		
Week 2	1.23 ± 0.86	1.66 ± 0.75		
Week 4	1.18 ± 0.72	1.61 ± 0.79		
Change (W4 – baseline)				0.83
Worsened	6 (13.6%)	4 (9.3%)		
No change	21 (47.7%)	20 (46.5%)		
Improved	17 (38.6%)	19 (44.2%)		
*Cramps*				
Mean symptom score ± SD			0.65 [0.31 - 1.35]	0.25
Baseline	0.71 ± 0.85	0.32 ± 0.64		
Week 2	0.36 ± 0.72	0.23 ± 0.36		
Week 4	0.43 ± 0.79	0.14 ± 0.35		
Change (W4 – baseline)				0.02
Decreased	17 (38.6%)	7 (16.3%)		
No change	21 (47.7%)	33 (76.7%)		
Increased	6 (13.6%)	3 (7.0%)		
*Flatulence*				
Mean symptom score ± SD			0.87 [0.62 - 1.22]	0.42
Baseline	0.86 ± 0.80	0.63 ± 0.73		
Week 2	0.64 ± 0.75	0.70 ± 0.74		
Week 4	0.96 ± 0.91	0.61 ± 0.66		
Change (W4 – baseline)				1.00
Decreased	10 (22.7%)	11 (25.6%)		
No change	23 (52.3%)	22 (51.2%)		
Increased	11 (25.0%)	10 (23.3%)		
*Anal irritation*				
Mean symptom score ± SD			0.33 [0.11 - 1.02]	0.055
Baseline	0.80 ± 1.11	0.54 ± 0.96		
Week 2	0.18 ± 0.54	0.09 ± 0.37		
Week 4	0.27 ± 0.73	0.02 ± 0.15		
Change (W4 – baseline)				0.45
Decreased	15 (34.1%)	12 (27.9%)		
No change	26 (59.1%)	30 (69.8%)		
Increased	3 (6.8%)	1 (2.3%)		

### Safety

Over the course of the study, 55 treatment-emergent adverse events (TEAEs) were reported in 26 children in the lactulose group (59.1%) and 80 TEAEs reported in 27 children in the PEG 4000 group (61.4%). These TEAEs are summarised in Table 4. The most frequently reported TEAEs were signs of local irritation of the anus and upper respiratory tract infections and related terms (rhinitis, pharyngitis, sinusitis, otitis media). The nature and incidence of individual TEAEs was similar in the two treatment groups. The majority of these events were considered mild and none were considered severe.

**Table 4:** Treatment-emergent adverse events (TEAEs) reported during the course of the study by treatment group (safety population; N = 88)

	**Lactulose**	**PEG 4000**
	**(N = 44)**	**(N = 44)**
Any TEAE*	26 (59.1%) [55 events]	27 (61.4%) [80 events]
Anal dilation	10 (22.7%) [14 events]	14 (31.8%) [11 events]
Upper respiratory tract infections	9 (20.5%) [11 events]	9 (20.5%) [11 events]
Anal fissure	5 (11.4%) [6 events]	9 (20.5%) [10 events]
Faecaloma	7 (15.9%) [10 events]	5 (11.4%) [6 events]
Hard faeces	4 (9.1%) [4 events]	3 (6.8%) [3 events]
Anal skin tags	1 (2.3%) [2 events]	5 (11.4%) [5 events]
Rhinorrhoea	1 (2.3%) [1 event]	3 (6.8%) [3 events]
Vomiting	None	3 (6.8%) [3 events]
Mild TEAEs	26 (59.1%) [53 events]	26 (59.1%) [72 events]
Moderate TEAEs	1 (2.3%) [2 events]	5 (11.4%) [8 events]
Severe TEAEs	None	None
TEAEs possibly or probably related to treatment	1 (2.3%) [2 events]	3 (6.8%) [3 events]
Serious TEAEs	1 (2.3%) [1 event]	2 (4.6%) [2 events]
TEAEs leading to death	None	None
TEAEs leading to treatment discontinuation	None	2 (4.6%) [5 events]

Data are presented as the number of patients (%), with the number of events given in square brackets. *Only individual events reported in more than two patients in either group are listed.

Five adverse events were considered possibly or probably related to the study drug, two in the lactulose group (diarrhoea and fever, both documented in the same infant) and three in the PEG 4000 group (three cases of diarrhoea). Two subjects, both in the PEG 4000 group, experienced TEAEs which led to permanent discontinuation of the study drug. These cases consisted of one case of vomiting and diarrhoea and one case of fever and vomiting associated with sinusitis. These two children were subsequently lost to follow-up and withdrawn from the study.

Three serious adverse events leading to hospitalisation were documented. One infant in the lactulose group experienced a varicella infection, one in the PEG 4000 group a pneumonia infection and a second in the PEG 4000 group a road traffic accident. Treatment was temporarily suspended during hospitalisation in the two children with infections. None of these three serious adverse events were considered related to the study medication. No deaths occurred during the course of the study.

Vital signs recorded at each study visit as well as the physical examination were comparable for the two study groups.

## Discussion

This study performed in Thailand showed PEG 4000 to be more efficacious than lactulose in increasing stool frequency in young children with chronic constipation and to be well-tolerated. This finding adds to the growing evidence base that PEG osmotic laxatives are more effective than lactulose in the treatment of constipation in children [10,26]. In terms of safety, the tolerability of PEG 4000 was satisfactory and broadly comparable to that of lactulose. Both treatments were well accepted and no clinically relevant safety issue was identified.

These findings are consistent with those of a large Chinese study in 216 older children aged from eight to eighteen years which also demonstrated greater efficacy of PEG 4000 compared to lactulose [17,18]. A meta-analysis published by the Cochrane collaboration estimated the on-treatment difference in stool frequency between patients receiving PEG preparations and those receiving lactulose to be 1.09 stools per week [95% CI: 0.02 to 2.17] [26]. Our findings (on-treatment intergroup difference of 0.3 stools/day) are towards the upper end of the range of the estimate of the meta-analysis.

The results of our study also complement those of a previous one evaluating the safety of PEG 4000 and lactulose in 96 children aged from six months to three years, performed in France [16]. This study demonstrated the good long-term safety of PEG 4000, and our findings are consistent with this. Efficacy was a secondary outcome in the French study, which found both PEG 4000 and lactulose to be effective in relieving constipation with greater improvements observed in the PEG 4000 group with respect to stool consistency, appetite, new-onset faecal impaction and recourse to enema use. As in this French study, we were also able to demonstrate a benefit of PEG 4000 over lactulose with respect to stool consistency and ease of stool passage, although not with respect to associated symptoms. In the meta-analysis published by the Cochrane collaboration [26], minor adverse events occurred with similar frequency in children treated with PEG preparations and with lactulose.

The study has a number of strengths and weaknesses. The strengths include the randomised, double-blind comparative design, which has not been used extensively in studies of constipation in paediatric populations, the qualification of the reference centres and the low rate of study discontinuations and of major protocol violations. Since the two preparations compared in this study have a different taste, there was some risk of compromising the blinding, although the medication was provided in identical sachets, using an identical dosing regimen. The mean treatment exposure was high in both groups and the acceptability of the two preparations was high, with compliance superior to >80% in all but three patients in both treatment arms. Data collected using a patient diary filled in by the parents cannot be independently ascertained, which may compromise their accuracy. However, the use of a patient diary represents a pragmatic solution to data collection in the community setting and such patient-related outcome measures are recommended in current guidelines for follow-up assessment of bowel habits in children [27]. In the PP population, but not in the ITT population, there was an indication of an interaction between treatment and study centre, with the treatment effect being more pronounced in patients treated at site 1 compared to site 2. However, it should be noted that the change from baseline in stool frequency at site 2 was still nearly twofold higher in the PEG 4000 group than in the lactulose group, and that the study was insufficiently powered to detect significant between-group differences at the site level. We have no obvious explanation for the treatment × site interaction in the PP population, but this may possibly relate to the fact that four of the five major protocol violations in the lactulose group related to patients enrolled at site 2.

The choice of inclusion criteria in this study merits some comment. At the time the study protocol was being drawn up, the Rome III criteria for infants [28] had not been published. We used a modification of the Rome II criteria for functional constipation in infants and preschool children (scybalous, pebble-like, hard stools for a majority of stools or firm stools two or less times/week, and no evidence of structural, endocrine, or metabolic disease present for at least two weeks). We modified the duration criterion to three months since we did not feel that treatment of very young children for one month with an experimental treatment could be justified if the constipation could be transient or self-resolving. A faecal incontinency criterion was added since this is frequently associated with functional constipation in children. However, in the event, no children were included on the basis of faecal incontinence alone. A longer minimum duration of symptoms and inclusion of a faecal incontinence criterion are present in the current Rome III criterion [28].

The superiority of PEG preparations over lactulose or other osmotic laxatives demonstrated in this and other studies and the limited tolerability of stimulant laxatives in children confer a favourable benefit-risk relationship on such preparations. This underlines the recommendations of current practice guidelines in which PEG preparations are identified as first-line treatment options. The NASPGHAN guidelines [27,29] recommend use of mineral oil (a lubricant) or magnesium hydroxide, lactulose, sorbitol or PEG (osmotic laxatives), or a combination of lubricant and laxative, and state that PEG appears to be superior to other osmotic agents in palatability and acceptance by children. The guidelines of the National Institute for Health and Clinical Excellence (NICE) identify PEG/electrolyte solutions as the recommended first-line treatment [30]. Effective treatment of children with constipation is important both to relieve discomfort and distress and to improve quality of life for patients and their parents. In addition, effective early management of constipation reduces the risk of persistence into adolescence and adulthood [7,8], thus reducing the overall burden and cost of disease from a public health perspective. For this reasons, the availability of effective and well-tolerated osmotic laxatives such as PEG 4000 have an important place in the management of chronic constipation in young children. A recent Cochrane review [26] concluded that PEG preparations may be superior to placebo, lactulose and magnesium hydroxide for the management of childhood constipation, and was associated with a lower incidence of adverse events.

## Conclusion

This randomised, double-blind comparative study provides robust and reliable evidence for the superior efficacy of PEG 4000 over lactulose in the treatment of chronic constipation in young children. The good safety and acceptability of PEG 4000 make it a first-line treatment of choice for young children in order to restore normal bowel habits.

## Competing interests

PG and HMF are employees of IPSEN, purveyors of the PEG 4000 preparation evaluated in this study. ST, NS, WV, PP and YT declare that they have no competing interests.

## Authors’ contributions

ST and NS are the principal investigators of the study. WV participated in the study design. PP and YT enrolled and evaluated the patients. PG and HMF initiated and funded the study, and supervised data collection and analyses. All authors read and approved the final manuscript.

## Pre-publication history

The pre-publication history for this paper can be accessed here:

http://www.biomedcentral.com/1471-2431/14/153/prepub
